# Isolation and characterization of the *Staphylococcus aureus* bacteriophage vB_SauS_SA2

**DOI:** 10.3934/microbiol.2019.3.285

**Published:** 2019-09-27

**Authors:** Jia Wang, Feiyang Zhao, Huzhi Sun, Qian Wang, Can Zhang, Wenhua Liu, Ling Zou, Qiang Pan, Huiying Ren

**Affiliations:** 1 Qingdao Agricultural University, College of Veterinary Medicine, Shandong 266109, China; 2 Qingdao Phagepharm Bio-tech Co, Ltd, Shandong 266109, China

**Keywords:** *Staphylococcus aureus*, Bacteriophage vB_SauS_SA2, Genome analysis

## Abstract

A novel bacteriophage vB_SauS_SA2 (hereafter designated SA2) that infects *Staphylococcus aureus* was isolated. At a multiplicity of infection (MOI) of 0.1, phage SA2 had a latent period of about 10 min with a burst size of 293 PFUs/infected cell (PFU, plaque forming unit). Phage SA2 had a double-stranded DNA genome with a length of 89,055 bp and a G + C content of 31.9%. The genome contained 130 open reading frames (ORFs), 28 of which had assigned functions, and 18 were unique. One tRNA gene (*tRNA^Asn^*) was discovered, and no virulence genes were identified. Its genome showed very low similarity with phage genomes deposited in public databases (75% nucleotide identity and 7% query coverage). The unique characteristics of phage SA2 led to the proposal of a new *Siphoviridae* genus named ‘SA2likevirus’.

## 1. Introduction

*Staphylococcus aureus* is an important prevalent pathogen that can cause a variety of infectious diseases in both humans and animals through different pathways [1],[2]. The multifarious diseases caused by *S. aureus* include suppurative infections, pneumonia, pericarditis and meningitis in humans [3] and various local or systemic infectious diseases in animals, such as avian arthritis, bovine mastitis and septicemia [4]. Though antibiotics have been widely used for many years, there is still an increasing number of infectious diseases. Especially, multiple drug-resistant strains of *S. aureus* increase rapidly, such as methicillin-resistant (MRSA) and vancomycin-resistant *S. aureus* (VRSA) strains [5],[6]. The inefficient treatment of bacterial infections cause substantial economic loss and has been a challenging issue in veterinary medicine. Therefore, it's of great significance to develop new therapies that can supplement or replace the use of antibiotics.

Bacteriophages (phages) are the most abundant and diverse biological entities on the planet, and the number of phages is estimated to be about 10^31^, approximately10 times of the number of host bacteria [7],[8]. Phages can affect the structure and function of microbial communities, and they play a key role in determining microbial diversity [9]. There are two common types of phages, i.e., lysogenic and lytic phages, and lytic phages can replicate, reproduce and release lysin and have high lytic activity [10]. Recently, phages have been recognized as natural, safe, highly specific and effective alternatives to antibiotics in preventing and treating bacterial infections caused by *S. aureus*, and they can be used either alone or in combination with other agents [11],[12].

In this study, we performed genome sequencing and biological characterization of *S. aureus* phage vB_SauS_SA2 isolated from sewage in a livestock market and proposed a new genus *Siphoviridae* called ‘SA2likevirus’.

## 2. Materials and methods

### 2.1. Bacterial strains and growth conditions

Fifty-three staphylococcal strains were isolated from the skin surface of several animals (pigs, rabbits, and chickens), and they belonged to seven different species, including *Staphylococcus aureus*, *S. saprophyticus*, *S. gallinarum*, *S. cohnii*, *S. sciuri*, *S. lentus* and *S. xylosus* (Table 1). All strains were cultivated in Luria-Bertani (LB) broth (Biocorp) at 37 °C. Stock cultures were stored in LB broth supplemented with 30% glycerol at −80 °C.

**Table 1.** Host range of phage vB_SauS_SA2.

No.	Strains	Efficiency of plating (EOP)	No.	Strains	Efficiency of plating (EOP)
1	*S. aureus* F2 (host)	1	28	*S. saprophyticus* A30	-
2	*S. aureus* ZTB1-5	1.034	29	*S. saprophyticus* C3	-
3	*S. aureus* PMJ 7-2	0.862	30	*S. saprophyticus* E2	-
4	*S. aureus* F3	0.079	31	*S. saprophyticus* F1	-
5	*S. aureus* TTB2-4	0.038	32	*S. gallinarum* A9	-
6	*S. aureus* F4	0.036	33	*S. gallinarum* A10	-
7	*S. aureus* TV2-2	0.025	34	*S. gallinarum* A21	-
8	*S. saprophyticus* E3	0.967	35	*S. gallinarum* A25	-
9	*S. saprophyticus* A26	0.931	36	*S. gallinarum* A31	-
10	*S. saprophyticus* A32	0.097	37	*S. cohnii* A17	-
11	*S. saprophyticus* A28	0.048	38	*S. cohnii* A18	-
12	*S. saprophyticus* A24	0.042	39	*S. cohnii* A23	-
13	*S. aureus* ZNZ 2-3	-	40	*S. cohnii* F6	-
14	*S. aureus* C1-4	-	41	*S. sciuri* E1	-
15	*S. aureus* A13	-	42	*S. sciuri* A3	-
16	*S. aureus* A20	-	43	*S. sciuri* F7	-
17	*S. aureus* A27	-	44	*S. lentus* JTB1-3	-
18	*S. aureus* F8	-	45	*S. lentus* A1	-
19	*S. aureus* PX-1	-	46	*S. lentus* F5	-
20	*S. aureus* TTB1-1	-	47	*S. lentus* C4	-
21	*S. aureus* TTB1-3	-	48	*S. lentus* E8	-
22	*S. aureus* TTB2-1	-	49	*S. lentus* C5	-
23	*S. aureus* STB1-3	-	50	*S. lentus* WZ-1	-
24	*S. aureus* ZTB1-3	-	51	*S. xylosus* A7	-
25	*S. saprophyticus* A4	-	52	*S. xylosus* E6	-
26	*S. saprophyticus* A15	-	53	*S. xylosus* A12	-
27	*S. saprophyticus* A29	-			

Notes: ‘−’ indicates that no plaques were observed

### 2.2. Phage isolation

Sewage samples (20 mL) were collected from a livestock market in Qingdao, Shandong province, China and filtered through a 0.22 µm membrane for sterilization. Phages were isolated from sewage using the conventional double-layer agar method [13]. Briefly, the filtrate was incubated with *S. aureus* in LB broth overnight at 37 °C. The culture broth was centrifuged at 12,000 × g for 10 min, and the supernatant was collected and filtered with a 0.22 µm membrane to remove bacterial residues. Then the supernatant was serially diluted in LB broth. Aliquots (100 µL) of these diluted phage suspensions, together with 100 µL of *S. aureus* culture, were mixed with 5 mL of soft top agar and poured on top of the solidified LB agar plates. The plates were incubated overnight at 37 °C to form plaques. Phage purification was repeated at least three times, and the final purified phages were then collected and stored at 4 °C.

### 2.3. Transmission electron microscopy (TEM)

The morphology of phage SA2 was examined by transmission electron microscopy (TEM) [14]. The phage suspension was added onto the surface of a copper grid and adsorbed for 15 min. The phages were negatively stained with 2% phosphotungstic acid in darkness for 10 min. The morphology of the phages was examined with a transmission electron microscope (HT7700, Japan) at 80 kV.

### 2.4. Host range

The host range of phage SA2 on the staphylococcal strains were determined using the efficiency of plating (EOP) [15]. The mixture of phage SA2 and the tested bacterial strains (Table 1) were incubated overnight at 37 °C, and the titers were determined using the double-layer agar method. The efficiency of plating (EOP) values were determined by calculating the ratio of PFUs of each phage-susceptible strain to PFUs obtained with *S. aureus* F2 strain. The experiment was repeated three times.

### 2.5. Thermal and pH stability, UV sensitivity

To determine the thermostability of phage SA2, the phage suspensions were incubated at various temperatures (40, 50, 60, 70 and 80 °C), and aliquots (100 µl) were collected after 20, 40, and 60 minutes of incubation, respectively. To evaluate the stability of the phages at different pH levels, the purified phages were incubated in LB broth at different pH levels ranging from 2 to 14 for 1, 2, 3 h, respectively. To observe the ultraviolet (UV) sensitivity of phage SA2, the phage suspensions were continuously exposed for 2 hours at 1.5 cm under an LED UV lamp (power 30 W, light intensity 26.23 µw/cm^2^). The aliquots were collected each 10 min post exposure. Phage samples were titered using the double-layer agar method [13]. Each experiment was performed in triplicate.

### 2.6. One-step growth curve

The one-step growth experiment of phage SA2 was carried out as described previously with minor modifications [16]. Briefly, the phages (1.67 × 10^8^ pfu/mL) were mixed with the *S. aureu*s F2 culture (1.01 × 10^9^ cfu/mL) at a MOI of 0.1 and incubated at 37 °C for 5 min. The suspension was centrifuged at 10,000 rpm for 30 s, and the pellets were re-suspended in LB broth, followed by incubation at 37 °C with shaking at 160 rpm. Aliquots (100 µL) were taken every 5 min within the first hour, every 20 minutes within the second hour and every 30 minutes within the third and fourth hours, respectively. The aliquots were then centrifuged at 13,000 g for 3 min, and the titers of phages in the supernatants were immediately determined using the double-layer agar method. The experiments were carried out in triplicates. The burst size was calculated as the ratio of the final count of liberated phage particles to the initial count of phage particles.

### 2.7. In vitro bacteriolytic activity

The *in vitro* bacteriolytic activity of phage SA2 was tested based on the absorbance (OD_630_) of the culture broth measured at 630 nm using spectrophotometry (ELX800, USA) [17]. The phages (2.8 × 10^8^ pfu/mL) were mixed with the *S. aureus* F2 culture (5.5 × 10^8^ cfu/mL) at different MOIs of 1, 0.1, 0.01, 0.001, 0.0001, and 0.00001, respectively, followed by incubation at 37 °C for 24 hours. *S. aureus* culture and LB broth served as a positive control and a negative control, respectively. The absorbance (OD_630_) of the culture broth was measured at 0.5, 1, 1.5, 2, 2.5, 3, 3.5, 4, 5, 6, 7, 8, 9, 10, 11, 12 and 24 h after the onset of incubation, respectively. The bacteriolytic activity was calculated at the corresponding time points. The experiment was performed in triplicate.

### 2.8. Phage genome extraction

Phage genomic DNA was extracted using the phenol-chloroform method [18]. In Brief, the phage suspension was firstly treated with RNase A (5 µg/mL) and DNase I (2 U/mL) at 37 °C for 30 min, followed by incubation at 80 °C for 15 min to deactivate DNase I. Then, purified phages were treated with proteinase K (50 µg/mL) at 56 °C for 1h, in the presence of SDS (0.5%) and EDTA (20 mM). The mixtures were mixed with an equal volume of phenol/chloroform/isoamyl alcohol (25:24:1), followed by centrifugation at 12,000 g for 20 min at 4 °C. The supernatant was mixed with an equal volume of isopropanol and kept at −20 °C overnight. After centrifugation, the pellets were washed three times with cold 75% ethanol. Finally, the pellets were air-dried, dissolved in 50 µl of TE buffer (10 mM Tris-HCl; 1 mM EDTA, pH 8) and stored at −20 °C.

### 2.9. Sulfate-polyacrylamide gel electrophoresis (SDS-PAGE)

The structural proteins of phage SA2 were analyzed by SDS-PAGE [19]. Following concentration with PEG 8000/NaCl, the phage particles were mixed with an equal volume of sample buffer (0.05 M Tris-HCl, pH 6.8; 10% glycerol, 2% SDS, 0.1% bromophenol blue, 1.56% DL-Dithiothreitol), and were heated in a boiling water bath for 10 min. After centrifugation (12,000 g, 10 min), the proteins in the supernatant were separated on a 12% SDS-PAGE and protein bands were visualized after staining with Coomassie brilliant blue. Protein bands were excised from the gel, digested by proteases and analyzed by Maldi mass spectrometry (Sangon Biotech, Shanghai).

### 2.10. Nature of the phage

To determine whether phage SA2 is lysogenic or lytic, suspicious lysogens were isolated as described with some modifications [20]. Briefly, phage SA2 was mixed with the host strain at MOI of 5 and incubated at 37 °C for 5 min and plated out. Ten colonies were randomly picked out, streaked out 2 times and incubated in LB broth overnight at 37 °C, and then extracted the bacterial genomes for phage gene detection by PCR. The isolates were mixed with phage SA2 and the sensitivity to phage SA2 was tested by the double-layer agar method.

Three primer pairs were designed to amplify 537 bp of the holin (SA2-hol), and 1443 bp of the lysin (SA2-lys) of phage SA2, respectively. The each primer sequences of phage SA2 was: SA2-hol-F 5′-GGGCATATGATGGCAGAATCAAAGAAACAG 3′; SA2-hol-R 5′-CAGCTCGAGTCATTGATTATCTTCCCCTTT 3′; SA2-lys-F 5′-CAGAAAGGGGAAGATAAT 3′; SA2-lys-R 5′-TGTAACGCCAATACCAAT 3′. The PCR amplification reactions were performed in 25 µL, including 1 µL of 30 ng of DNA template, 2 µL of 25 µM of each primer, 12.5 µL of 2 × TS INGKE Master Mix and 9.5 µL ddH_2_O. The reaction condition was as follows: pre-denaturation at 94 °C for 5 min, followed by 30 cycles of denaturation at 94 °C for 1 min, annealing at 55 °C for 1 min, extension at 72 °C for 2 min, and final extension at 72 °C for 10 min. The PCR amplified products were analyzed by electrophoresis on a 1.0% agarose gel.

### 2.11. Genome sequence analysis

The complete sequence was annotated using the subsystem technology (RAST, http://rast.nmpdr.org) and GeneMark (http://opal.biology.gatech.edu/GeneMark/) [21]. All predicted open reading frames (ORFs) were verified using the online BLASTP (http://www.ncbi.nlm.nih.gov/BLAST). The putative transfer RNA (tRNA)-encoding genes were searched using tRNAscan-SE (http://trna.ucsc.edu/tRNAscan-SE/) [22]. The circular view of the genomic alignment of phage SA2 with other staphylococcal phages was constructed using BLAST Ring Image Generator (BRIG) [23], and the multiple sequences were aligned using Mauve (http://darlinglab.org/mauve/mauve.html) for comparative genomic analysis [24]. The phylogenetic tree of phage SA2 was constructed based on the large terminator subunit, the major capsid, and DNA polymerase using the ClustalW program in MEGA 6 [25].

### 2.12. Nucleotide sequence accession number

The complete genome sequence of phage SA2 had been deposited in the GenBank database under the accession number MH356730.

### 2.13. Statistical analysis

All data were expressed as mean ± standard deviation (SD) and analyzed using GraphPad Prism 6.0.

## 3. Results and discussion

### 3.1. Morphology of phage SA2

**Figure 1.**
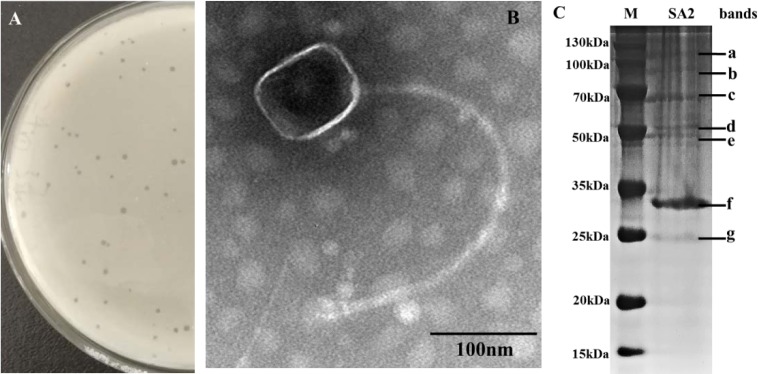
Characterization of phage vB_SauS_SA2. (A) Phage plaques formed on double-layered agar plate, (B) TEM image of phage vB_SauS_SA2 (magnification: × 40.0 K), (C) SDS-PAGE showing the structural proteins of phage vB_SauS_SA2, lane M: protein marker.

The *S. aureus* phage SA2 was isolated from sewage using the double-layer agar method with *S. aureus* F2 strain. The plaques of phage SA2 were clear with a diameter of about 1 mm (Figure 1A). The TEM image showed that phage SA2 had a polyhedral head (75 nm in diameter) and a long curved tail (410 nm in length) (Figure 1B). According to the current classification system developed by the International Committee on Taxonomy of Viruses, phage SA2 was classified as a member of the family *Siphoviridae* of the order *Caudovirales*. This phage was designated vB_SauS_SA2, in accordance with the recommendation proposed by Kropinski et al. [26].

### 3.2. Characterization of phage structural proteins

The structural proteins of phage SA2 were separated by SDS-PAGE, and seven protein bands were visualized on the gel (Figure 1C). The protein band of about 33 kDa (band f in Figure 1C) was identified to be the major capsid protein by mass spectrometry, which is relatively smaller than the capsid protein (42 kDa) of phage SA2. In order to verify the sequenced genome, the gene of the major capsid protein was amplified by polymerase chain reaction using primer (F: 5′-ACAACAGAAGGTGCATCAGC-3′; R: 5′-AACGACAAAAGTCTTCCCAG-3′), and amplification products were sequenced by Shanghai Personal Biotech Co., Ltd. (Personalbio, China), and it revealed that the aligned sequence was consistent with the annotated sequence. Therefore, the smaller size of the separated capsid protein was possibly caused by protein degradation. In addition, the other six protein bands were not detected by mass spectrometry due to their very low concentrations.

### 3.3. Host range

The host range of phage SA2 was determined by assessing its ability to form plaques on lawns of fifty-three Staphylococcus strains (Table 1). The results showed that phage SA2 lysed 23% (12/53) of test strains. Seven *S. aureus* strains and five *S. saprophyticus* strains were sensitive to phage SA2, and no correlation was observed between lytic activity and isolated animal species. Our spot test experiment showed that phage SA2 could lyse 14 test strains and the high sensitivity of the spot test may lead to some false positive results. The difference between EOP results and spot test results has been reported in many literatures [15]. *S. aureus* phages can lyse a variety of Staphylococcus species, for example, vB_SauM-fRuSau02 can lyse *S. saprophyticus*, *S. intermedius*, and other Staphylococcus species [27]. Like *S. aureus* phage vB_SauM-fRuSau02, SA2 could lyse Staphylococcus species (*S. aureus* and *S. saprophyticus*).

**Figure 2.**
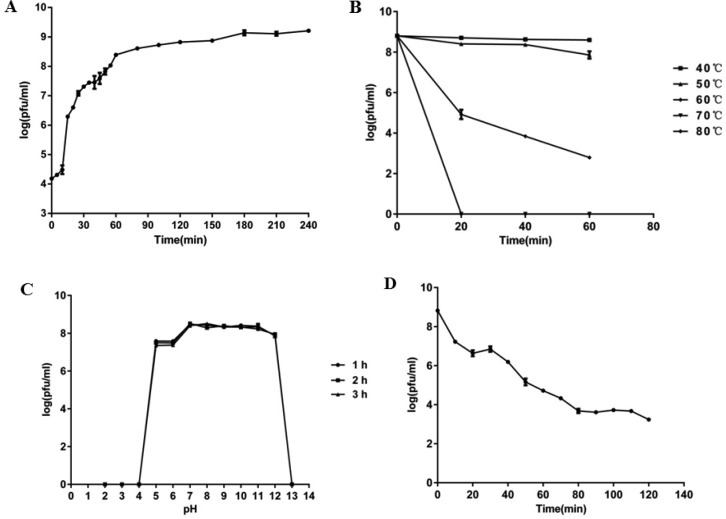
One-step growth curve (A), thermal stability (B), pH stability (C) and UV sensitivity (D) of phage vB_SauS_SA2. Data are expressed as mean ± SD.

### 3.4. Biological characteristics of SA2

The one-step growth curve showed that the latent period and burst period of phage SA2 were 10 min and 170 min, respectively, and the burst size was about 293 PFUs/infected cell (Figure 2A). The positive linear relationship was found between lysis time and burst size, which is similar to other isogenic λ-phages [28]. Phage SA2 was stable at temperatures ranging from 40 to 50 °C, but the phage titer decreased significantly after heat treatment (60 °C, 20 minutes) and the phages were completely inactivated at 70 °C (Figure 2B). The phage activity was relatively stable over a broad pH range (7–12) after at least 3 h of incubation, but under extreme pH conditions (below pH 5 or above pH 12) the phage titer declined abruptly (Figure 2C). After exposure to ultraviolet light, the phage titer decreased significantly by five orders of magnitude within 2 hours (Figure 2D), showing that UV had a certain lethal effect on the phage SA2.

### 3.5. In vitro bacteriolytic activity

**Figure 3.**
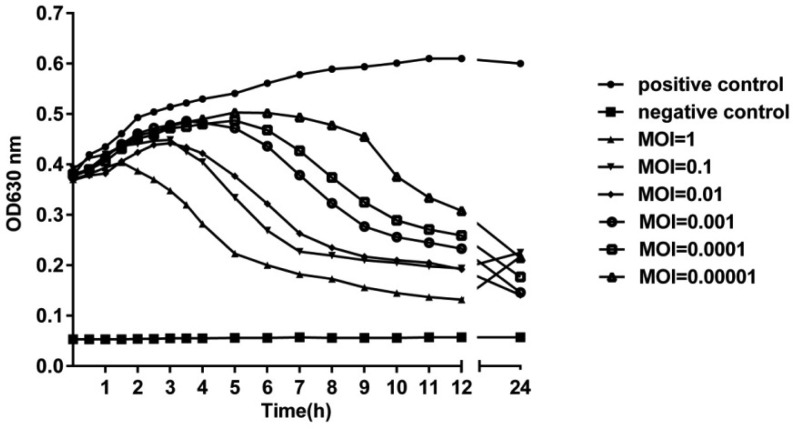
In vitro bacteriolytic activity of phage vB_SauS_SA2. The absorbance of the culture broth containing phage SA2 and *S. aureus* at different MOIs increased gradually during the first few hours and then decreased remarkably. At 24 h, most of bacteria were lysed by phage SA2 at all MOIs. Data are expressed as mean ± SD.

The in vitro bacteriolytic activity of phage SA2 was determined using spectrophotometry, as shown in Figure 3. The absorbance of the positive control increased continuously within 24 hours, while the absorbance of the negative control remained unchanged. In contrast, the absorbance of the culture broth containing phage SA2 and *S. aureus* at different MOIs increased gradually during the first few hours and then decreased remarkably, indicating that *S. aureus* was lysed by phage SA2. At 24 h, most of the bacteria were lysed by phage SA2 at all MOIs. The decrease in absorbance during the first few hours (3–7 hours) depended on the MOI values. The higher the MOI value is, the faster the absorbance decreases. For example, the absorbance started to decline as early as about 2 h after the onset of co-incubation at a MOI of 1.

### 3.6. Characterization of phage SA2 genome

**Figure 4.**
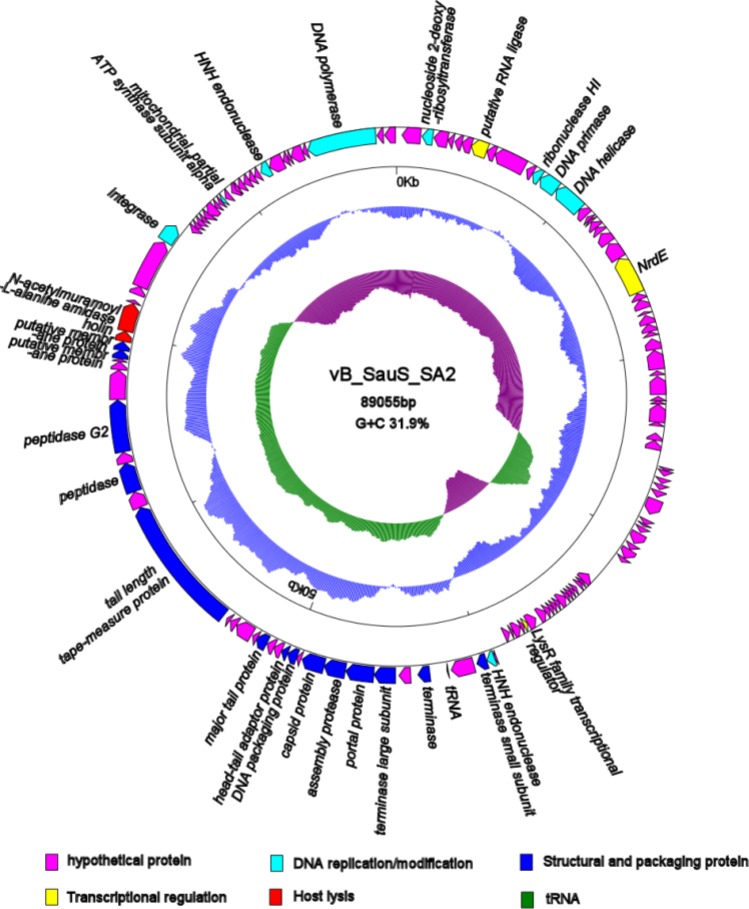
Genome structure of phage vB_SauS_SA2. The arrows indicate the direction of transcription of each gene. The two innermost circles represent GC-skew ((G-C)/(G+C)) and GC contents.

Analysis of the whole genome showed that SA2 was a linear double-stranded DNA molecule of 89,055 bp with an average G + C content of 31.9%. The genome contained 130 predicted open reading frames (ORFs), and the inverted ORFs accounted for 62.3% (81 ORFs) of the total genome. The majority of the ORFs presented an ATG start codon (88.5%), while seven started with TTG, eight with GTG. One tRNA gene (*tRNA^Asn^*) was discovered in the genome of phage SA2. No virulence gene was detected. Of the 130 ORFs, 28 had assigned functions, 18 were unique and similar genes were not found in the deposited genomes in the GenBank, while the remaining ORFs were annotated as hypothetical proteins (Table 2). Phage SA2 possessed the same modular genomic architecture as the majority of dsDNA phages [29] (Figure 4), including DNA replication and modification, transcriptional regulation, phage packaging and structural proteins, and proteins involved in host lysis.

**Table 2.** ORF analysis of the vB_SauS_SA2 genome.

ORFs	Strand	Start	Stop	Size(aa)	Start codon	Function	Best-match BLASTp Result	Identities	E-values	No.in Genbank
ORF1	-	1304	291	337	ATG	hypothetical protein	*Bacteroides finegoldii*	27/96(28%)	5.1	WP_007759001.1
ORF2	-	1967	1386	193	ATG	nucleoside 2-deoxyribosyltransferase	Staphylococcus phage vB_Sau_S24	157/201(78%)	3.00E-109	ARM69542.1
ORF3	-	2790	2035	251	ATG	hypothetical protein	Staphylococcus phage vB_SscM-1	171/252(68%)	1.00E-118	ANT44727.1
ORF4	-	3105	2806	99	ATG	hypothetical protein	Edhazardia aedis USNM 41457	21/62(34%)	3.10E-01	EJW04541.1
ORF5	-	3574	3185	129	ATG	hypothetical protein	*Staphylococcus gallinarum*	78/131(60%)	3.00E-37	WP_107591340.1
ORF6	-	4104	3571	177	ATG	hypothetical protein	Staphylococcus phage 6ec	91/143(64%)	7.00E-60	YP_009042597.1
ORF7	-	4988	4104	294	ATG	putative RNA ligase	Staphylococcus phage S25-3	236/294(80%)	2.00E-166	YP_008854188.1
ORF8	-	5469	5002	155	ATG	hypothetical protein	*Staphylococcus sp*. ZWU0021	49/124(40%)	2.00E-16	WP_047504225.1
ORF9	-	7217	5481	578	ATG	hypothetical protein	Staphylococcus phage 6ec	303/587(52%)	0	YP_009042601.1
ORF10	-	7758	7375	127	TTG	hypothetical protein	*Beggiatoa leptomitoformis*	38/95(40%)	1E-13	WP_062147175.1
ORF11	-	8242	7769	157	ATG	ribonuclease HI	*Candidatus Rokubacteria bacterium*	63/149(42%)	5.00E-31	PYM77762.1
ORF12	-	9315	8257	352	TTG	DNA primase	Staphylococcus phage vB_SepS_SEP9	197/348(57%)	5.00E-135	YP_009007737.1
ORF13	-	10968	9328	546	ATG	DNA helicase	Staphylococcus phage 6ec	330/548(60%)	0	YP_009042604.1
ORF14	-	11471	10968	167	ATG	hypothetical protein	Staphylococcus phage 6ec	75/163(46%)	1.00E-31	YP_009042606.1
ORF15	-	11760	11560	66	ATG	-	-			
ORF16	-	11896	11753	47	GTG	hypothetical protein	Staphylococcus phage 6ec	24/45(53%)	8.00E-07	YP_009042609.1
ORF17	-	12314	11919	131	ATG	hypothetical protein	Staphylococcus phage 6ec	54/135(40%	1.00E-23	YP_009042610.1
ORF18	-	12657	12301	118	ATG	hypothetical protein	Staphylococcus phage 6ec	58/117(50%)	9.00E-29	YP_009042611.1
ORF19	-	13367	12729	212	ATG	-	-			
ORF20	-	14382	13426	318	ATG	hypothetical protein	Staphylococcus phage 6ec	245/318(77%)	0	YP_009042612.1
ORF21	-	16501	14408	697	TTG	NrdE	Staphylococcus phage 6ec	596/697(86%	0	YP_009042613.1
ORF22	-	16894	16538	118	ATG	hypothetical protein	Staphylococcus phage 6ec	64/114(56%)	1.00E-39	YP_009042614.1
ORF23	-	17415	16909	168	ATG	hypothetical protein	*Staphylococcus sp.* HMSC078A12	32/99(32%)	1e-04	WP_070843680.1
ORF24	-	17732	17589	47	ATG	hypothetical protein	*Clostridiales bacterium* GWE2_32_10	17/36(47%)	3.2	OGO86757.1
ORF25	-	18286	17804	160	ATG	hypothetical protein	*Streptomyces pini*	26/66(39%)	3.8	WP_093847145.1
ORF26	-	18707	18288	139	ATG	-	-			
ORF27	-	18961	18722	79	ATG	hypothetical protein	*Colwellia sp.* 12G3	17/51(33%)	5.6	WP_101231934.1
ORF28	-	19706	19107	199	ATG	hypothetical protein	Staphylococcus phage vB_SauM-fRuSau02	46/162(28%)	3.00E-07	AST15717.1
ORF29	-	20789	19740	349	ATG	hypothetical protein	Staphylococcus phage vB_SepS_SEP9	256/349(73%)	0	YP_009007759.1
ORF30	-	21101	20865	78	ATG	hypothetical protein	Staphylococcus phage 6ec	46/77(60%)	1.00E-24	YP_009042628.1
ORF31	-	22151	21159	330	ATG	hypothetical protein	Staphylococcus phage 6ec	211/319(66%)	7.00E-146	YP_009042630.1
ORF32	-	22492	22253	79	ATG	hypothetical protein	Staphylococcus phage vB_SepS_SEP9	29/77(38%)	4.00E-12	YP_009007765.1
ORF33	-	22674	22492	60	ATG	hypothetical protein	*Hyphomicrobium sp.* 99	17/46(37%)	7.7	WP_045836444.1
ORF34	-	23656	22679	325	ATG	hypothetical protein	Staphylococcus phage vB_SepS_SEP9	207/327(63%)	1.00E-150	YP_009007766.1
ORF35	-	23856	23656	66	ATG	hypothetical protein	*Staphylococcus*	32/58(55%)	1.00E-15	WP_000460098.1
ORF36	-	24630	24238	130	ATG	-	-			
ORF37	-	25133	24645	162	ATG	-	-			
ORF38	+	25939	26070	43	TTG	-	-			
ORF39	+	26084	26365	93	ATG	hypothetical protein	Staphylococcus phage SA3	60/94(64%)	2.00E-34	ASZ78147.1
ORF40	+	26501	26761	86	ATG	hypothetical protein	Staphylococcus phage vB_Sau_Clo6	81/86(94%)	2.00E-48	ARM69284.1
ORF41	+	26823	27083	86	ATG	hypothetical protein	Staphylococcus phage 6ec	54/83(65%)	3.00E-33	YP_009042635.1
ORF42	+	27164	27496	110	ATG	hypothetical protein	Staphylococcus phage vB_Sau_S24	102/108(94%)	3.00E-69	ARM69501.1
ORF43	+	27632	28432	266	ATG	hypothetical protein	Staphylococcus phage vB_SepS_SEP9	38/95(40%)	3.00E-09	YP_009007769.1
ORF44	+	28755	29003	82	ATG	hypothetical protein	Staphylococcus phage phiSA_BS1	25/65(38%)	0.43	AVP40361.1
ORF45	+	29004	29123	39	ATG	-	-			
ORF46	+	29201	29467	88	ATG	-	-			
ORF47	+	29542	30114	190	ATG	hypothetical protein	Staphylococcus phage vB_SepS_SEP9	81/194(42%)	5.00E-29	YP_009007774.1
ORF48	+	30195	30488	97	ATG	-	-			
ORF49	+	30502	30996	164	ATG	hypothetical protein	Staphylococcus phage VB_SavM_JYL01	113/159(71%)	7.00E-70	AXU40178.1
ORF50	+	31022	31252	76	ATG	hypothetical protein	Notothenia coriiceps	16/44(36%)	3.50E+00	XP_010776353.1
ORF51	-	33303	32833	156	ATG	-	-			
ORF52	-	33453	33316	45	ATG	-	-			
ORF53	-	33658	33446	70	ATG	hypothetical protein	*Staphylococcus equorum*	41/64(64%)	2.00E-19	WP_069816167.1
ORF54	-	33998	33804	64	ATG	hypothetical protein	Eubacterium aggregans	19/48(40%)	6.00E+00	SDZ94007.1
ORF55	-	34196	34029	55	ATG	-	-			
ORF56	-	34397	34197	66	ATG	hypothetical protein	*Staphylococcus cohnii*	38/63(60%)	2.00E-18	WP_107504991.1
ORF57	-	34592	34398	64	ATG	hypothetical protein	*Staphylococcus petrasii*	39/62(63%)	6.00E-19	WP_103297505.1
ORF58	-	35027	34605	140	ATG	hypothetical protein	*Staphylococcus pasteuri*	120/133(90%)	3.00E-83	WP_072291777.1
ORF59	-	35247	35029	72	ATG	hypothetical protein	*Staphylococcus saprophyticus*	43/70(61%)	4.00E-18	WP_069822260.1
ORF60	-	35472	35272	66	ATG	-	-			
ORF61	-	35680	35477	67	ATG	hypothetical protein	*Staphylococcus xylosus*	47/66(71%)	2.00E-28	WP_107562606.1
ORF62	-	35936	35682	84	ATG	hypothetical protein	*Staphylococcus xylosus*	60/78(77%)	3.00E-36	WP_017722706.1
ORF63	-	36377	35937	146	GTG	hypothetical protein	*Staphylococcus saprophyticus*	57/83(69%)	8.00E-25	WP_069878080.1
ORF64	-	37092	36574	172	ATG	hypothetical protein	Staphylococcus phage 6ec	69/170(41%)	6.00E-31	YP_009042510.1
ORF65	-	37318	37103	71	ATG	LysR familytranscriptional regulator	*Vibrio coralliilyticus*	21/46(46%)	5.90E+00	WP_095664875.1
ORF66	-	37521	37327	64	ATG	hypothetical protein	Staphylococcus phage 6ec	38/63(60%)	2.00E-20	YP_009042511.1
ORF67	-	37979	37536	147	ATG	hypothetical protein	Staphylococcus phage vB_SepS_SEP9	72/147(49%)	3.00E-44	YP_009007788.1
ORF68	-	38133	38008	41	ATG	-	-			
ORF69	-	38521	38195	108	ATG	hypothetical protein	Staphylococcus phage vB_SepS_SEP9	67/107(63%)	4.00E-43	YP_009007791.1
ORF70	+	39341	39463	40	ATG	hypothetical protein	Staphylococcus phage vB_SepS_SEP9	27/40(68%)	9.00E-12	YP_009007664.1
ORF71	+	39444	39854	136	ATG	HNH endonuclease	Staphylococcus phage vB_SepS_SEP9	96/133(72%)	6.00E-69	YP_009007665.1
ORF72	+	39873	40394	173	ATG	terminase small subunit	Staphylococcus phage 6ec	115/169(68%)	2.00E-80	YP_009042522.1
ORF73	+	40551	41732	393	ATG	hypothetical protein	Staphylococcus phage 6ec	264/392(67%)	0	YP_009042524.1
ORF74	+	42831	43448	205	ATG	terminase	*Bacillus cereus*	109/193(56%)	1.00E-75	WP_098666102.1
ORF75	+	43813	44409	198	ATG	hypothetical protein	*Clostridium chromiireducens*	49/101(49%)	2.00E-15	WP_079438120.1
ORF76	+	44582	45673	363	ATG	terminase large subunit	Staphylococcus phage 6ec	299/362(83%)	0.00E+00	YP_009042527.1
ORF77	+	45686	47119	477	GTG	portal protein	Staphylococcus phage vB_SepS_SEP9	331/464(71%)	0	YP_009007672.1
ORF78	+	47144	48253	369	ATG	assembly protease	Staphylococcus phage 6ec	265/377(70%)	0.00E+00	YP_009042529.1
ORF79	+	48253	49419	388	ATG	capsid protein	Staphylococcus phage PMBT8	305/383(80%)	0	QDF14302.1
ORF80	+	49476	49703	75	ATG	hypothetical protein	Staphylococcus phage vB_SepS_SEP9	40/69(58%)	2.00E-17	YP_009007675.1
ORF81	+	49704	50213	169	ATG	DNA packaging protein	Staphylococcus phage 6ec	112/167(67%)	4.00E-78	YP_009042532.1
ORF82	+	50213	50551	112	ATG	head-tail adaptor protein	Staphylococcus phage vB_SepS_SEP9	72/108(67%)	1.00E-49	YP_009007677.1
ORF83	+	50551	50970	139	ATG	hypothetical protein	Staphylococcus phage vB_SepS_SEP9	95/138(69%)	2.00E-63	YP_009007678.1
ORF84	+	50967	51362	131	ATG	hypothetical protein	Staphylococcus phage vB_SepS_SEP9	98/127(77%)	3.00E-69	YP_009007679.1
ORF85	+	51387	51983	198	ATG	major tail protein	Staphylococcus phage vB_SepS_SEP9	169/197(86%)	5.00E-117	YP_009007680.1
ORF86	+	51937	52200	87	TTG	-	-			
ORF87	+	52271	53170	299	ATG	hypothetical protein	*Enterococcus faecalis*	146/292(50%)	1.00E-99	WP_048943754.1
ORF88	+	53201	53551	116	ATG	hypothetical protein	Staphylococcus phage vB_SepS_SEP9	78/112(70%)	4.00E-47	YP_009007681.1
ORF89	+	53593	53778	61	ATG	hypothetical protein	Staphylococcus phage vB_SepS_SEP9	42/65(65%)	3.00E-19	YP_009007682.1
ORF90	+	53999	60997	2332	ATG	tail length tape-measure protein	Staphylococcus phage vB_SepS_SEP9	1271/2473(51%)	0	YP_009007684.1
ORF91	+	61012	61845	277	TTG	hypothetical protein	Staphylococcus phage vB_SepS_SEP9	167/278(60%)	8.00E-117	YP_009007685.1
ORF92	+	61856	63382	508	ATG	peptidase	*Staphylococcus sp.* HMSC071G07	251/529(47%)	3.00E-167	WP_070503109.1
ORF93	+	63375	63944	189	ATG	hypothetical protein	*Staphylococcus sp.* LCT-H4	117/189(62%)	3.00E-85	WP_071331791.1
ORF94	+	63962	66619	885	ATG	peptidase G2	*Staphylococcus sp.* LCT-H4	714/885(81%)	0	WP_071331792.1
ORF95	+	66638	68143	501	ATG	hypothetical protein	*Staphylococcus sp.* LCT-H4	361/500(72%)	0	WP_071331793.1
ORF96	+	68158	68574	138	ATG	hypothetical protein	*Staphylococcus cohnii*	72/125(58%)	7.00E-46	WP_046467806.1
ORF97	+	68576	68719	47	ATG	hypothetical protein	Staphylococcus phage IME1354_01	38/47(81%)	3.00E-20	ARM68393.1
ORF98	+	68729	69181	150	ATG	putative membrane protein	Staphylococcus phage vB_SepS_SEP9	69/143(48%)	9.00E-41	YP_009007694.1
ORF99	+	69151	69603	150	GTG	putative membrane protein	Staphylococcus phage vB_SepS_SEP9	83/137(61%)	1.00E-56	YP_009007695.1
ORF100	+	69620	70156	178	ATG	holin	Staphylococcus phage phiIPLA-RODI	88/141(62%)	2.00E-55	YP_009195894.1
ORF101	+	70153	71595	480	ATG	N-acetylmuramoyl-L-alanine amidase	*Staphylococcus aureus* A9765	311/485(64%)	0	EFB99605.1
ORF102	+	71617	71790	57	ATG	hypothetical protein	Staphylococcus phage phiSA_BS1	29/57(51%)	2.00E-13	AVP40394.1
ORF103	+	72089	72526	145	TTG	hypothetical protein	Staphylococcus phage vB_SscM-1	69/135(51%)	4.00E-41	ANT44707.1
ORF104	+	72516	75095	859	ATG	hypothetical protein	*Staphylococcus cohnii*	348/490(71%)	0.00E+00	WP_052722067.1
ORF105	+	75155	76129	324	ATG	integrase	Staphylococcus phage 6ec	221/326(68%)	1.00E-159	YP_009042560.1
ORF106	-	76698	76378	106	ATG	hypothetical protein	Staphylococcus phage vB_SepS_SEP9	68/106(64%)	4.00E-41	YP_009007701.1
ORF107	-	76933	76688	81	ATG	hypothetical protein	Staphylococcus phage vB_SepS_SEP9	38/75(51%)	1.00E-18	YP_009007702.1
ORF108	-	77195	76923	90	ATG	hypothetical protein	Staphylococcus phage vB_SepS_SEP9	56/88(64%)	3.00E-32	YP_009007703.1
ORF109	-	77409	77215	64	ATG	hypothetical protein	Staphylococcus phage 6ec	19/55(35%)	4.80E-01	YP_009042564.1
ORF110	-	77684	77409	91	ATG	-	-			
ORF111	-	78219	77677	180	ATG	hypothetical protein	Staphylococcus phage vB_SepS_SEP9	106/176(60%)	2.00E-71	YP_009007706.1
ORF112	-	78455	78231	74	ATG	hypothetical protein	*Hymenobacter sp.* CCM 8763	25/74(34%)	7.00E-07	WP_116942204.1
ORF113	-	78702	78517	61	GTG	hypothetical protein	Staphylococcus phage JD007	33/58(57%)	3.00E-11	YP_007112904.1
ORF114	-	78887	78708	59	ATG	ATP synthase subunitalpha, mitochondrial, partial	Trichinella zimbabwensis	17/46(37%)	8.30E+00	KRZ02401.1
ORF115	-	79271	78963	102	GTG	hypothetical protein	*Staphylococcus gallinarum*	36/67(54%)	2.00E-06	WP_042740106.1
ORF116	-	79649	79278	123	GTG	hypothetical protein	*Staphylococcus sp.* ZWU0021	77/124(62%)	2.00E-41	WP_052190986.1
ORF117	-	79878	79678	66	ATG	-	-			
ORF118	-	80212	79880	110	ATG	hypothetical protein	Staphylococcus phage phiSA_BS2	52/108(48%)	9.00E-27	AVR55465.1
ORF119	-	80582	80247	111	ATG	hypothetical protein	Staphylococcus phage phiSA_BS2	97/111(87%)	3.00E-66	AVR55465.1
ORF120	-	80916	80587	109	ATG	hypothetical protein	Staphylococcus phage phiSA_BS2	99/109(91%)	3.00E-64	AVR55466.1
ORF121	-	81234	80947	95	ATG	hypothetical protein	Staphylococcus phage 6ec	67/91(74%)	6.00E-40	YP_009042576.1
ORF122	-	81856	81332	174	GTG	HNH endonuclease	*Gilliamella apicola*	26/76(34%)	3.1	WP_086317515.1
ORF123	-	82684	81884	266	ATG	hypothetical protein	Staphylococcus phage vB_Sau_S24	236/262(90%)	4.00E-172	ARM69552.1
ORF124	-	82983	82702	93	ATG	hypothetical protein	*Oceanobacillus massiliensis*	25/56(45%)	1.80E+00	WP_010651149.1
ORF125	-	83165	83001	54	ATG	hypothetical protein	*Clostridium perfringens*	28/54(52%)	2.00E-06	WP_110035294.1
ORF126	-	83858	83187	223	ATG	hypothetical protein	Staphylococcus phage vB_SepS_SEP9	132/220(60%)	1.00E-96	YP_009007717.1
ORF127	-	84112	83861	83	ATG	-	-			
ORF128	-	87927	84157	1256	ATG	DNA polymerase	Staphylococcus phage vB_SepS_SEP9	879/1245(71%)	0	YP_009007719.1
ORF129	-	88344	87997	115	ATG	hypothetical protein	Staphylococcus phage 6ec	41/110(37%)	1.00E-09	YP_009042587.1
ORF130	-	88995	88411	194	ATG	hypothetical protein	Staphylococcus phage 6ec	110/192(57%)	5.00E-64	YP_009042588.1

DNA replication and modification modules are involved in the coordinated activity of several enzymes. A nucleoside 2-deoxyribosyltransferase was encoded by ORF2 of phage SA2 that showed 78% homology to Staphylococcus phage vB_Sau_S24 (*Myoviridae*). ORF11 was predicted to encode ribonuclease HI, an enzyme that cleaves the RNA strand of an RNA/DNA hybrid, and it had 42% homology to the genes of Candidatus Rokubacteria bacteria, but without homology to any phage. Other replication proteins of phage SA2 were similar to the proteins of *Staphylococcus epidermidis* phage. DNA primase (encoded by ORF12) can be used to initiate DNA synthesis [30]. DNA helicase (encoded by ORF13) can utilize ATP hydrolysis to separate the DNA double helix into individual strands [31]. The main function of DNA polymerase (encoded by ORF128) is to fill DNA gaps generated during DNA repair, recombination, and replication. ORF71 and ORF122 were predicted to encode HNH endonucleases that play a variety of roles in the phage lifecycle [32]. These proteins are commonly observed in phages of the *Twortlikevirus* genus of the family *Herelleviridae* (formerly the subfamily Spounavirinae of the family *Myoviridae*), but they are rarely found in staphylococcal *Siphoviridae* phages [20].

ORF7 was predicted to encode a putative RNA ligase that exhibits 80% high sequence identity to *Staphylococcus* phage S25-3. ORF21 encoded NrdE, an aerobic Ib ribonucleotide reductase with the basic function of reducing ribonucleotides to deoxyribonucleotides [33]. The LysR family of transcriptional regulators (encoded by ORF65) regulates a diverse set of genes, including those involved in virulence, metabolism, quorum sensing and motor genes [34].

ORF72 and ORF76 encoded the small terminase subunit and large terminase subunit, respectively, which resembled the packaging module of Staphylococcus phage 6ec. Terminase is an enzyme that can insert a single viral genome into the interior of a viral procapsid by a process known as ‘encapsulation or packaging’, and it consists of a small subunit and a large subunit [35]. Typically, the small terminase subunit specifically recognizes viral DNA and recruits the large terminase protein for the initial cleavage. The large terminase subunit has an ATPase activity that provides energy for packaging initiation and termination [36],[37]. Portal protein (encoded by ORF77) shares high amino acid sequence similarity with Staphylococcus phage vB_SepS_SEP9, which can inject DNA into the host cell through a pathway formed by portal protein [38]. Assembly protease (encoded by ORF78) and DNA packaging protein (encoded by ORF81) were also found in phage SA2.

Among the phage structural proteins, ORF82 was predicted to be a head-tail adaptor protein that acted as an adaptor and bound directly to portal proteins during connector assembly [39]. ORF79 and ORF85 were predicted to encode a capsid protein and a major tail protein, respectively. ORF90 was predicted to encode a tail length tape-measure protein (TMP), and TMP was the longest protein product with 2,332 amino acids. TMP determines the length of the tail, and the length of the corresponding gene is proportional to the tail [40], which may explain the large size of the SA2 tail (410 nm). This protein contained peptidoglycan hydrolytic domains (an N-terminal lytic transglycosylase SLT domain and a C-terminal peptidase_M23 domain), which is similar to other staphylococcal Siphoviruses proteins [41]. It is possible that a long tail/TMP may be beneficial in locating phage for optimal infection, ultimately docking the phage (tail tube) on the cell membrane in a more efficient manner [40].

ORF92 was predicted as a peptidase with a prophage endopeptidase tail, which may belong to virion-associated peptidoglycan hydrolase (VAPGHs). VAPGHs are structural components of phage that locally degrade peptidoglycans of the bacterial cell walls during infection [42]. ORF94 was predicted to encode a peptidase G2 with a pectate lyase superfamily protein that could be involved in the degradation of extracellular polymers [20].

The lysis cassette of phage SA2 was comprised of holin and N-acetylmuramoyl-L-alanine amidase. Holin (encoded by ORF100) is a small phage encoding protein that forms large holes on the cell membrane to alter the permeability and performs similar functions as signal peptides [43]. It can be categorized into three subtypes: class I, class II and class III [44]. The holin protein of phage SA2 had two potential class II membrane-spanning domains and shared 62% identity with Staphylococcus phage phiIPLA-RODI (*Myoviridae*). ORF101 was predicted to encode N-acetylmuramoyl-L-alanine amidase and contained three domains: SH3 peptidoglycan-binding domain, PGRP super-family conserved domain and N-terminal CHAP endopeptidase domain. N-acetylmuramoyl- L -alanine amidase is specifically dedicated to lysis, and holin dedicates to the amidase activation at a precisely defined time [45], which can be attributed to the function of endolysin. Phage likely lyses the host cell to release its progeny through the holin-endolysin lytic system, which is thought to be universal in almost all dsDNA phages [46],[47].

ORF105 was predicted to encode integrase. The results showed that 36 of the 130 ORFs of phage SA2 were homologous to bacterial genome (28%), indicating that these bacteria could contain temperate phages. Our experiments indicated that phage SA2 was able to lysogenize the host bacteria, and ORF100 (holin) (Figure S1A) and ORF101 (lysin) (Figure S1B) of phage SA2 could be detected by PCR in the three isolated colonies, and they were not more sensitive to phage SA2. Interestingly, the integrase gene was also found in the genomic sequence of phage SEP9 (YP_009007700.1), which had 68% homology with SA2, but phage SEP9 was not a lysogenic phage.

### 3.7. Phylogenetic and comparative genomic analysis

**Figure 5.**
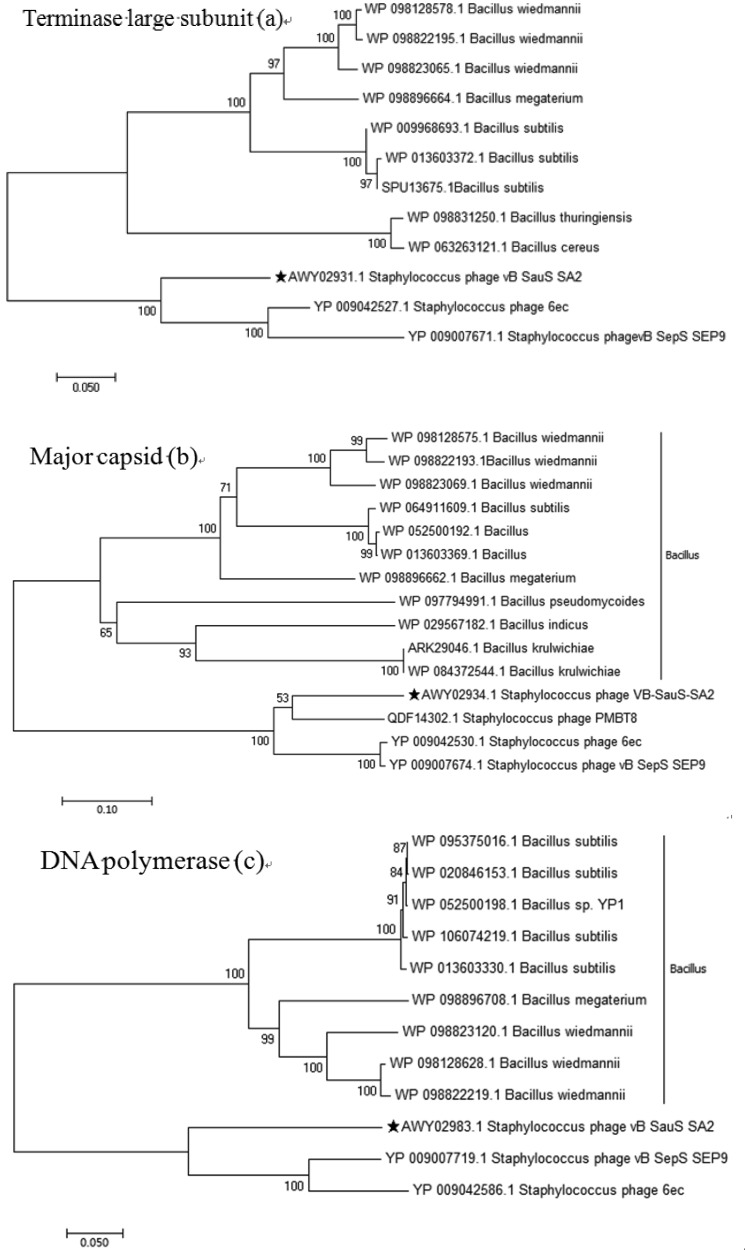
Phylogenetic analysis of the phage vB_SauS_SA2. The amino acid sequences of the terminase large subunit (a), major capsid (b), DNA polymerase (c) were compared using MEGA6, and phylogenetic tree was generated using the neighbour-joining method and 1000 bootstrap replicates.

Genome-wide BLAST analysis revealed that the SA2 genome showed very low similarity with phage genomes deposited in public databases (Table S1). The sequence of phage SA2 was 75% homologous to those of both staphylococcal phage 6ec (KJ804259.1) and staphylococcal phage vB_SepS_SEP9 (KF929199.1), while genome coverage was only 7% and 5%, respectively (Figure S2). Multi-genomic alignment revealed that SA2 had similar regions with homology to the 6ec and SEP9 genomes, but their locations differed (Figure S3). The phylogenetic tree indicated that phage SA2 was related to *S. epidermidis* bacteriophages 6ec and SEP9, but differed from other staphylococcal phages (Figure 5). The comparative genomic analysis showed that the genome of phage SA2 was 89.5 kb, similar to those of phage SEP9 (92.4 kb), phage 6ec (93.8 kb) and phage PMBT8 (88.1 kb), and the overall G+C content was 31.9%, similar to those of phage SEP9 (29.6%), phage 6ec (29.3%) and phage PMBT8 (31.6%). The phage SEP9 belongs to a ‘Sep9likevirus’ genus, and the phage 6ec has not been assigned to an exact genus yet. So far, most members of the *Siphoviridae* family of bacteriophages remain unclassified. Since the morphological and genomic of phage SA2 was different from ‘3alikevirus’, ‘77likevirus’, ‘Phietalike-virus’, and ‘Sep9likevirus’ of Staphylococcal *Siphoviruses* , a new *Siphoviridae* genus, named ‘SA2likevirus, was proposed based on the unique characteristics of phage SA2.

## 4. Conclusions

In this study, we isolated and characterized a novel lysogenic phage SA2 against *S. aureus*, which belonged to the family *Siphoviridae*. Phage SA2 showed lytic activity against several *S. aureus* strains and *S. saprophyticus* strains. At a MOI of 0.1, phage SA2 showed a short latent period and a long burst period, and the burst size was 293 PFUs/infected cell. Phage SA2 was stable over a wide pH range of 7–12 at 40–50 °C, but it was sensitive to ultraviolet light.

The phage SA2 had a polyhedral head (75 nm in diameter) and a long curved tail (400 nm in length), which was different from that of ‘3alikevirus’, ‘77likevirus’, ‘Phietalike-virus’, and ‘Sep9likevirus’ of Staphylococcal Siphoviruses. The phage SA2 genome showed very low similarity with all phage genomes deposited in public databases. Its linear dsDNA genome was comprised of 130 ORFs, 28 of which had assigned functions, and 18 were unique. One tRNA gene (*tRNA^Asn^*) was discovered, and no virulence genes were identified. Holin and N-acetylmuramoyl-L-alanine amidase were predicted, indicating that SA2 may be a newer therapeutic agent against *S. aureus* infection.

In conclusion, the *Siphoviridae* phage SA2 isolated in this study had the characteristics of a short latent period, a long burst period, and low genomic homology. It was different from any other known *Siphoviridae* phages, so we proposed a new *Siphoviridae* genus named ‘SA2likevirus’. Phage SA2 could lyse *S. aureus* strains and *S. saprophyticus* stains, and the unique ability to lyse host cells makes it possible to use phage SA2 as a new tool to explore the mechanisms of pathogenesis and resistance of multiple drug-resistant strains of *S. aureus*. The findings may provide a valuable reference for further development of phage-based biocontrol agents that are effective against *S. aureus*.
