# Supplementary material for: Isolation and characterization of the Staphylococcus aureus bacteriophage vB_SauS_SA2
Source: AIMS Microbiol. 2019 Sep 27;5(3):285–307. doi: 10.3934/microbiol.2019.3.285 (PMC6787349; doi:10.3934/microbiol.2019.3.285)
Supplement: Supplementary file 1 [file microbiol-05-03-285-s001.pdf]

Microbiology

http://www.aimspress.com/journal/microbiology

AIMS Microbiology, 5 (3): 285–307.
DOI: 10.3934/microbiol.2019.3.285
Received: 30 June 2019
Accepted: 25 September 2019
Published: 27 September 2019

Research article

Isolation and characterization of the *Staphylococcus aureus* bacteriophage vB_SauS_SA2

Jia Wang[1], Feiyang Zhao[1], Huzhi Sun[2], Qian Wang[1], Can Zhang[1], Wenhua Liu[1], Ling Zou[1], Qiang Pan[2] and Huiying Ren[1,*]

[1] Qingdao Agricultural University, College of Veterinary Medicine, Shandong 266109, China
[2] Qingdao Phagepharm Bio-tech Co, Ltd, Shandong 266109, China

* **Correspondence:** Email: renren0228@sina.com; Phone: +8653286080734; fax: +8653286080452.

Abstract: A novel bacteriophage vB_SauS_SA2 (hereafter designated SA2) that infects *Staphylococcus aureus* was isolated. At a multiplicity of infection (MOI) of 0.1, phage SA2 had a latent period of about 10 min with a burst size of 293 PFUs/infected cell (PFU, plaque forming unit). Phage SA2 had a double-stranded DNA genome with a length of 89,055 bp and a G+C content of 31.9%. The genome contained 130 open reading frames (ORFs), 28 of which had assigned functions, and 18 were unique. One tRNA gene ($tRNA^{Asn}$) was discovered, and no virulence genes were identified. Its genome showed very low similarity with phage genomes deposited in public databases (75% nucleotide identity and 7% query coverage). The unique characteristics of phage SA2 led to the proposal of a new *Siphoviridae* genus named 'SA2likevirus'.

Keywords: *Staphylococcus aureus*; bacteriophage vB_SauS_SA2; genome analysis

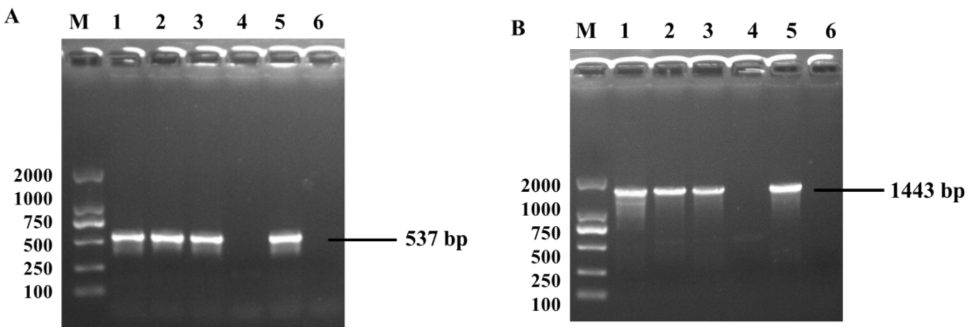

Figure S1. Nature of the phage vB_SauS_SA2. (A) The PCR product of ORF100 (holin), (B) The PCR product of ORF101 (lysin). Lane M: DL2000 DNA Marker, lane 1-3: lysogenic bacteria, lane 4: *S. aureus* F2 (host), lane 5: phage vB_SauS_SA2, lane 6: ddH_2O.

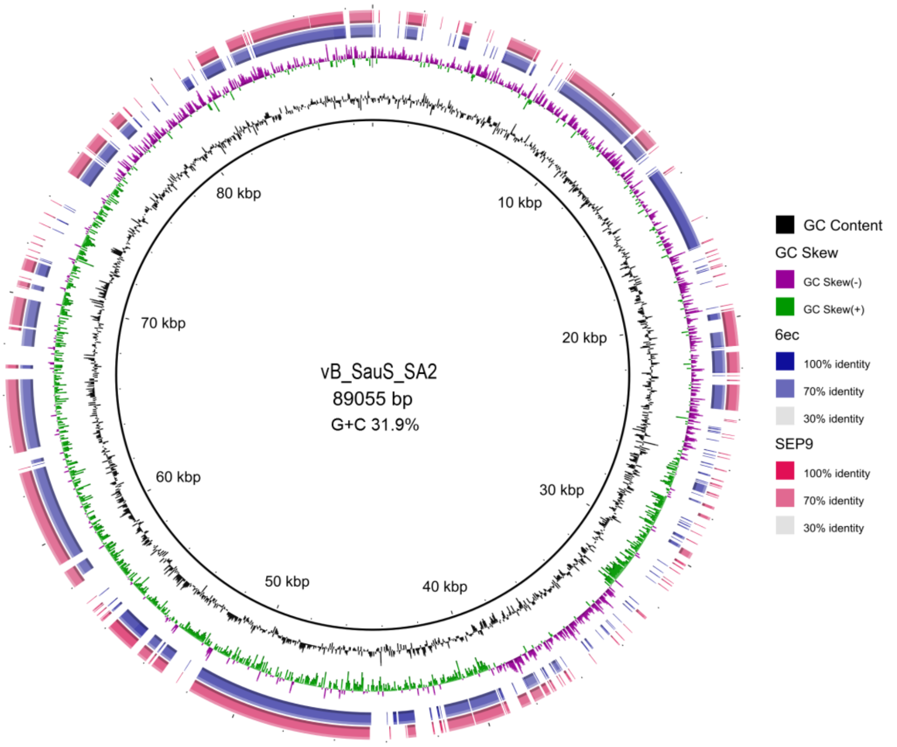

Figure S2. Circular view of the genomic alignment of phage vB_SauS_SA2 compared with phage 6ec and phage SEP9. The homology between phage SA2 and phage 6ec is shown in blue. The homology between phage SA2 and phage SEP9 is shown in red. The color intensity is proportional to the sequence homology. The two innermost circles represent GC contents and GC-skew [(G-C)/(G+C)].

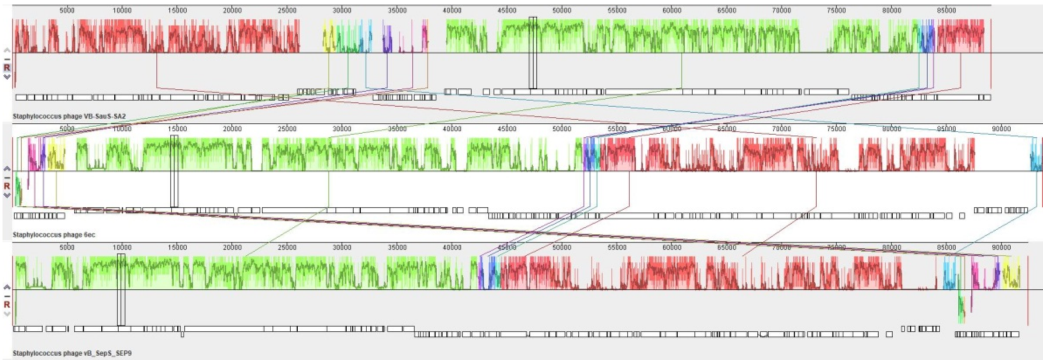

Figure S3. Comparative genome analysis of phage vB_SauS_SA2 with phage 6ec and phage SEP9. Lines connecting blocks are indicative of homologous regions among three phage genomes.

Table S1. vB_SauS_SA2 alignment result using BLAST program.

Accession	Description	Max. score	Total score	E value	Query cover (%)	Identity (%)
KJ804259.1	*Staphylococcus* phage 6ec, complete genome	1687	3571	0.0	7	75
KF929199.1	*Staphylococcus* phage vB_SepS_SEP9, complete genome	1676	2557	0.0	5	75
LR134264.1	*Staphylococcus hyicus* strain NCTC7944 genome assembly, chromosome: 1	1059	1059	0.0	1	78
CP022096.2	*Staphylococcus pettenkoferi* strain FDAARGOS_288 chromosome, complete genome	1020	1020	0.0	1	78
MH028956.1	*Staphylococcus* phage phiSA_BS2, complete genome	1009	1073	0.0	0	92
MF417890.1	*Uncultured Caudovirales* phage clone 7F_15, partial genome	985	1337	0.0	2	77
MF417946.1	*Uncultured Caudovirales* phage clone 7S_10, partial genome	976	1328	0.0	2	77
LN554884.1	*Staphylococcus xylosus* genome assembly *Staphylococcus xylosus* C2a, chromosome : Chr1	976	976	0.0	1	79
CP014113.2	*Staphylococcus saprophyticus* strain FDAARGOS_168 chromosome, complete genome	944	944	0.0	1	77
MK893987.1	*Staphylococcus* phage PMBT8, complete genome	900	2381	0.0	6	74

AIMS Press

© 2019 the Author(s), licensee AIMS Press. This is an open access article distributed under the terms of the Creative Commons Attribution License (http://creativecommons.org/licenses/by/4.0)
